# Strengthening the evidence-base of integrated care for people with multi-morbidity in Europe using Multi-Criteria Decision Analysis (MCDA)

**DOI:** 10.1186/s12913-018-3367-4

**Published:** 2018-07-24

**Authors:** Maureen Rutten-van Mölken, Fenna Leijten, Maaike Hoedemakers, Apostolos Tsiachristas, Nick Verbeek, Milad Karimi, Roland Bal, Antoinette de Bont, Kamrul Islam, Jan Erik Askildsen, Thomas Czypionka, Markus Kraus, Mirjana Huic, János György Pitter, Verena Vogt, Jonathan Stokes, Erik Baltaxe, on behalf of the SELFIE consortium

**Affiliations:** 1 School of Health Policy and Management, Erasmus University Rotterdam, Rotterdam, the Netherlands; 2 Institute for Medical Technology Assessment, Erasmus University Rotterdam, Rotterdam, the Netherlands; 3 Health Economics Research Centre, Nuffield Department of Population Health, University of Oxford, Oxford, UK; 4 Department of Economics, University of Bergen, Bergen, Norway; 5 Institute for Advanced Studies, Vienna, Austria; 6 Agency for Quality and Accreditation in Health Care and Social Welfare, Zagreb, Croatia; 7 Syreon Research Institute, Budapest, Hungary; 8 Department of Health Care Management, Technische Universität Berlin, Berlin, Germany; 9 Manchester Centre for Health Economics, Manchester Academic Health Science Centre, School of Health Sciences, University of Manchester, Manchester, UK; 10 Institut d’Investigacions Biomèdiques August Pi i Sunyer (IDIBAPS), Hospital Clinic de Barcelona, Universitat de Barcelona, Barcelona, Spain

**Keywords:** Integrated care, Multi-morbidity, Multi-criteria decision analysis, Economic evaluation, Triple aim, Outcomes, Cost

## Abstract

**Background:**

Evaluation of integrated care programmes for individuals with multi-morbidity requires a broader evaluation framework and a broader definition of added value than is common in cost-utility analysis. This is possible through the use of Multi-Criteria Decision Analysis (MCDA).

**Methods and results:**

This paper presents the seven steps of an MCDA to evaluate 17 different integrated care programmes for individuals with multi-morbidity in 8 European countries participating in the 4-year, EU-funded SELFIE project. In step one, qualitative research was undertaken to better understand the decision-context of these programmes. The programmes faced decisions related to their sustainability in terms of reimbursement, continuation, extension, and/or wider implementation. In step two, a uniform set of decision criteria was defined in terms of outcomes measured across the 17 programmes: physical functioning, psychological well-being, social relationships and participation, enjoyment of life, resilience, person-centeredness, continuity of care, and total health and social care costs. These were supplemented by programme-type specific outcomes. Step three presents the quasi-experimental studies designed to measure the performance of the programmes on the decision criteria. Step four gives details of the methods (Discrete Choice Experiment, Swing Weighting) to determine the relative importance of the decision criteria among five stakeholder groups per country. An example in step five illustrates the value-based method of MCDA by which the performance of the programmes on each decision criterion is combined with the weight of the respective criterion to derive an overall value score. Step six describes how we deal with uncertainty and introduces the Conditional Multi-Attribute Acceptability Curve. Step seven addresses the interpretation of results in stakeholder workshops.

**Discussion:**

By discussing our solutions to the challenges involved in creating a uniform MCDA approach for the evaluation of different programmes, this paper provides guidance to future evaluations and stimulates debate on how to evaluate integrated care for multi-morbidity.

**Electronic supplementary material:**

The online version of this article (10.1186/s12913-018-3367-4) contains supplementary material, which is available to authorized users.

## Background

With increasing life expectancy, the prevalence of multi-morbidity and the individual and socio-economic burden thereof is on the rise; this trend is seen worldwide [1–3]. Multi-morbidity is commonly defined as the co-occurrence of two or more chronic health conditions within one individual [4]. Conditions can co-exist for a number of reasons: they may share a common risk factor, be part of the same underlying disease-continuum, one disease may cause or increase the risk of the other or their co-existence may be random chance. Compared to people with single conditions, people with multi-morbidity have a lower life expectancy [5], a worse quality of life [6], higher healthcare utilization [7], and are more likely to be absent from work [8] and leave the workforce prematurely [9]. Multi-morbidity disproportionally affects people with lower socio-economic status; a Scottish study showed that the onset of multi-morbidity occurred 10–15 years earlier in people living in the most as compared to the least deprived areas [10]. Furthermore, people with multi-morbidity experience a greater burden of disease caused by the fragmentation in or duplication of services provided by multiple professionals working in different sectors mostly following single-disease guidelines [11, 12]. This may lead to conflicting treatment goals, unforeseen treatment interactions and overly demanding appeals on an individual’s self-management capability, which jeopardises compliance.

The provision of integrated care is increasingly seen as a means for addressing the complex needs of people with multi-morbidity. Recently, the World Health Organisation (WHO) has reinforced the importance of integration of care in its worldwide call for people-centred and integrated health services [13]. Various innovative programmes have been established internationally to provide integrated care to individuals with multi-morbidity [14–19]. Although attention for multi-morbidity is increasing, to date there is still too little research in this area [20], as a result of which the evidence of the effectiveness and cost-effectiveness of such programmes is relatively limited. This can be explained by the disease-specific focus of most research, the adoption of inadequate methodology to evaluate these complex interventions, the challenges associated with data collection and linkage, the inconsistent selection of outcome measures and the lack of multi-morbidity-specific outcome measures.

One of the aims of SELFIE, a large four-year European Horizon2020-funded project that started in September 2015 (See Table 1), is to strengthen the evidence-base of integrated care programmes for individuals with multi-morbidity by using a comprehensive evaluation approach called Multi-Criteria Decision Analysis (MCDA) [21, 22]. In SELFIE, eight countries, i.e., Austria, Croatia, Germany, Hungary, the Netherlands (coordinator), Norway, Spain, and the United Kingdom, are performing MCDAs of 17 promising integrated care programmes for multi-morbidity. The aim of this paper is to describe the methodological details of the MCDA approach applied in SELFIE by explaining the empirical study designs of the programmes, the development of a uniform set of outcome measures used in the MCDA evaluations, the weight-elicitation methods to determine the importance of the outcomes for the MCDA, and the uncertainty analysis. This paper can provide inspiration and guidance to future evaluations of integrated care programmes for multi-morbidity and stimulate international debate on how to comprehensively evaluate such programmes.

**Table 1:** About the SELFIE project

SELFIE (*S*ustainable int*E*grated chronic care mode*L*s for multi-morbidity: delivery, *FI*nancing, and performanc*E*) is a Horizon2020 funded EU project that aims to contribute to the improvement of person-centred care for persons with multi-morbidity by proposing evidence-based, economically sustainable, integrated care programmes that stimulate cooperation across health and social care and are supported by appropriate financing and payment schemes.	
More specifically, SELFIE aims to:	
• Develop a taxonomy of promising integrated care programmes for persons with multi-morbidity	
• Provide evidence-based advice on matching financing/payment schemes with adequate incentives to implement integrated care	
• Provide empirical evidence of the impact of promising integrated care on a wide range of outcomes using Multi-Criteria Decision Analysis	
• Develop implementation and change strategies tailored to different care settings and contexts in Europe, especially Central and Eastern Europe	
The SELFIE consortium includes eight organisations in the following countries: the Netherlands (coordinator), Austria, Croatia, Germany, Hungary, Norway, Spain, and the UK. https://www.selfie2020.eu [Grant Agreement No 634288]	

## Methods and results

In this section we describe the selection of the integrated care programmes, the general MCDA evaluation framework and the implementation of the seven steps of MCDA in the SELFIE project. The challenges involved in this implementation and the choices we made to overcome them are addressed in the discussion section.

### Programme selection

**Fig. 1:**
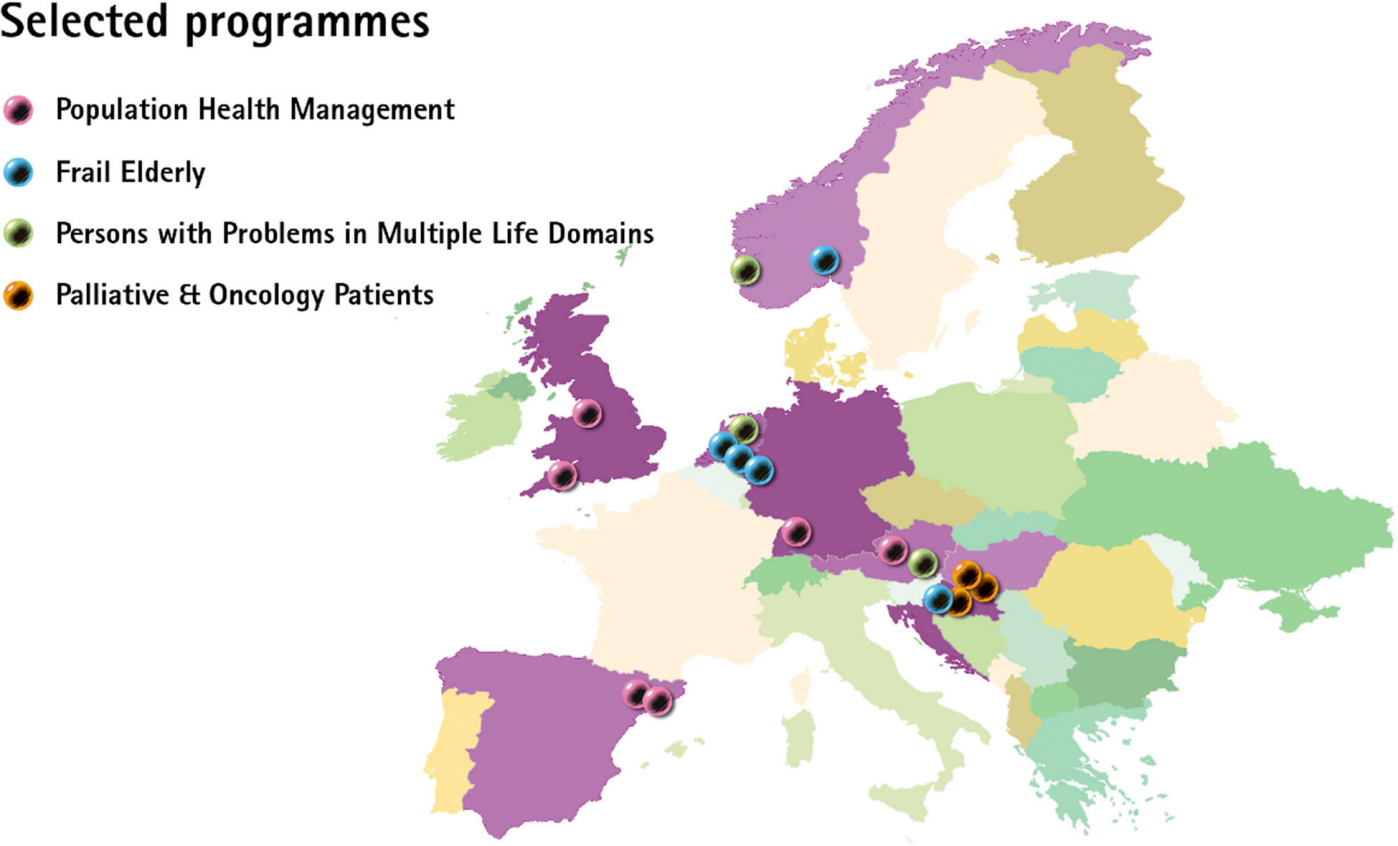
Geographic overview of selected 17 programmes

To identify promising candidate programmes, each country applied a search strategy using the findings from an international scoping review that was also conducted in the SELFIE project [19], national publications on previous and on-going programmes and projects, and consultation of national experts and networks. The final selection of two to three programmes per country was guided by a combination of scientific and pragmatic criteria. The primary scientific criteria focused on the care process itself, requiring that the programmes addressed multi-morbidity and met our operational definition of integrated care. Multi-morbidity was defined as at least two chronic conditions, physical or mental, occurring in one person at the same time, where one is not just a known complication of the other. Integrated care was defined as the structured efforts to provide coordinated, pro-active, person-centred, multidisciplinary care by two or more communicating and collaborating care providers that may work at the same organisation or different organisations, either within the healthcare or across the health, social, or community care sector (including informal care). We also gave priority to innovative programmes, i.e., bottom-up programmes with a clear goal and programmes in which individuals and informal caregivers had an active role, in which health- and social care were collaborating, and that focussed on continuity of care. Pragmatic selection criteria pertained to the availability or collectability of outcomes data, an on-going status of the programme for at least another two years, the transferability to other care settings, and the willingness to collaborate with the SELFIE project. Moreover, we aimed to have a variation across programmes with respect to their aims, target group, scope (e.g., small-scale case finding, screening, regional approaches, population health management), and focus (e.g., prevention, collaboration between health- and social care, palliative care, transfer care). The 17 programmes that were selected were grouped into four categories: 1) population health management programmes (*n* = 6), 2) frail elderly programmes (*n* = 5), 3) programmes for individuals at the end-of-life and oncology patients (*n* = 3), and 4) programmes for vulnerable individuals who face problems in multiple life domains, like health, housing, and financial problems (*n* = 3). Fig. 1 shows where the programmes are situated. They are further described in the section Measuring performance.

### Evaluation framework

The reason to opt for MCDA as an evaluation method stems from 1) the increased complexity of integrated care programmes when they target individuals with multiple morbidities [23] and 2) the need to adopt a more holistic, person-centred understanding of ‘value’ when evaluating the added benefit of these programmes. Regarding the first reason, integrated care programmes are considered to be complex, even if they focus on a single disease, because they commonly consist of a package of interacting interventions that intervene at different levels, i.e., they target individuals, providers, organisations, and/or sectors [23]. This is reinforced when the target population includes individuals with multi-morbidity. What adds to the complexity is that the programmes are tailored to the context in which they are implemented, and they interact with this context. During the dynamic implementation process, the programmes are continuously improved as more experience is gained. Furthermore, these programmes have a variety of intended outcomes at different levels, especially in multi-morbidity, and their effectiveness is impacted by the behaviour of those delivering and receiving the interventions. Regarding the second reason, we adopt a more holistic, person-centred understanding of ‘value’ because the standard cost-utility analysis in which a cost per QALY is calculated may be insufficient to capture the whole spectrum of relevant outcomes. Integrated care programmes, especially for individuals with multi-morbidity, do not only aim to improve health but also well-being, experience of care and efficiency. Sometimes the goal is just to align the services better and organise sufficient support to enable people to remain in control of their life. As a consequence, we seek to adopt an evaluation framework that is broad enough to incorporate a wide range of different outcomes, called ‘criteria’ in MCDA-terminology, to capture different components of the added value of these programmes [24, 25].

In SELFIE, we are using a multi-attribute value-based method of MCDA, which applies a weighting to the various outcomes of an integrated care programme and its comparator, from one or more perspectives, to calculate an overall value score [26]. In this type of MCDA, the performance of each integrated care programme and its comparator on all criteria are determined separately from the importance, or weights, of these criteria. For both the programme and the comparator, the weighted performance on each criterion is aggregated into an overall value score, which is then compared between the two. It was decided upfront that the MCDA method and the weights should be re-usable in the future. To facilitate this, we plan to create an online tool with the criteria weights from different perspectives (i.e., different groups of stakeholders). Others can use the tool to evaluate their own integrated care programmes in the future.

Seven steps are commonly undertaken in an MCDA: 1) establish the decision-context, 2) identify and structure criteria, 3) determine the performance on criteria, 4) determine the weights of the criteria, 5) create an overall value score, 6) perform sensitivity analyses, 7) interpret results [27]. Below we describe how we have applied these steps in SELFIE.

### Decision context (step 1)

To better understand the context of the selected programmes, we conducted qualitative research on each, including document analyses and interviews with programme-initiators, managers, representatives of payer organisations, care providers (physicians, nurses, social care staff), participants, and informal caregivers. This resulted in 17 ‘thick description’ reports (accessible via https://www.selfie2020.eu/). The thick descriptions were structured according to the components of a conceptual framework for integrated care for multi-morbidity that was developed at the beginning of the SELFIE project [28]. The individuals with multi-morbidity in their environment with their resources are the heart of the framework that is surrounded by the micro, meso, and macro layers of six components: 1) service delivery, 2) leadership and governance, 3) workforce, 4) financing, 5) technologies and medical products, and 6) information and research. Within these components, elements of integrated care that have previously been reported to contribute to its effectiveness are identified and described in the framework. In the thick descriptions, a formal description of the ‘hard facts’ in each component is given, as well as a description that goes one layer deeper and addresses the ‘soft facts’ that lay beneath the surface. The hard facts include for example the formal roles of the professionals involved, services provided, organisational structure, legal status, ICT support, and purchasing and payment contracts. The soft facts include for example the culture of the organisation, the extent to which there is a common vision, social relationships between staff members, management support, and power issues. The thick descriptions also systematically describe the barriers to the implementation of the programmes and strategies applied to overcome them. Furthermore, the thick descriptions reviewed existing evaluations of the programmes, most of which were methodologically weak. To enhance our understanding of the context in which the integrated care programmes are operating, the thick descriptions start with a macro-level description of the health and social care systems and policies in the country or region of interest.

The thick descriptions revealed that the decision-context that these programmes face is related to long-term sustainability in terms of reimbursement, continuation, extension, and/or wider implementation in their own region or country. Hence, the aim of the MCDA is to inform these decisions by comparing each of the 17 programmes to usual care.

An important part of understanding the decision context is identifying the stakeholders relevant to the decision-making process, whose value judgements will be included in the MCDA. The stakeholders considered relevant to inform the decision making surrounding integrated care for multi-morbidity are representatives of five groups (the 5P’s): Patients, Partners and other informal caregivers, Professionals, Payers, and Policy makers.

### Criteria (step 2)

The second step in an MCDA, is to identify and structure the decision criteria, which are the measures of performance of the programmes that are considered relevant to inform decision making. In SELFIE these are defined in terms of outcomes. We created a long-list of potentially relevant outcomes obtained from four sources: 1) a literature review, 2) national workshops with representatives from the 5P’s in the eight countries in the SELFIE project, 3) eight focus groups with individuals with multi-morbidity, one in each country [29], and 4) a review of outcomes currently being used in the 17 selected integrated care programmes. To support the process of selecting a feasible number of outcome measures, we clustered the outcomes into higher-level concepts and categorised them according to the Triple Aim, i.e., improving population health and well-being, improving experience with care, and reducing costs or cost-growth [30, 31]. The long-list was shortened to a core set of outcomes, a process that was guided by the following criteria:

- Relevance to multi-morbidity in different contexts and population groups;
- Relevance across the 17 integrated care programmes;
- Non-redundancy, i.e., there is little overlap between them;
- Preference independence, i.e., the weight of one outcome can be elicited independently from the performance score of another outcome;
- Operationalisability, e.g., preferring original, and widely accepted performance measures over self-constructed scales, avoiding proxies;
- Sensitivity to short-term intervention effect, i.e., the outcomes should be sensitive to the impact of a programme on newly enrolled individuals within a 12 to 24 month evaluation period.

Extensive discussions within the SELFIE consortium led to a consensus that we should focus on patient-reported outcome measures (PROMS) and patient-reported experience measures (PREMS). These PROMS and PREMS extend the list of structural indicators (e.g., the presence of an individual-portal, the use of a risk-prediction algorithm), process indicators (e.g., percentage of individuals with an individual care plan), and utilisation-based proxies of health outcomes (e.g., percentage of individuals admitted to hospital for a certain complication) that programmes are frequently using for monitoring and auditing purposes because they can easily be extracted from existing databases. We agreed that the set of outcome measures in our evaluations should go beyond clinical outcomes (e.g., HbA1c in diabetes), and should focus more broadly on well-being. Moreover, the outcomes that were frequently mentioned by individuals with multi-morbidity in the focus groups received high importance in the selection process, which eventually led to the core set of outcome measures shown in Table 2. This list is termed the core set, because it pertains to outcomes to be measured in each of the 17 SELFIE evaluations. The fact that the core set of outcome measures is not specific to a particular disease or programme enables the re-usability of the importance-weights in future evaluations (e.g., via the planned SELFIE online tool).

**Table 2:** Overview of the core set and programme-type specific outcomes in SELFIE

Outcomes for integrated care for individuals with multi-morbidity					
Triple Aim	Core set outcomes	Programme-type specific outcomes			
		Population health management	Frail elderly	Palliative and oncology	Problems in multiple life domains
Health & well-being	Physical functioning	Activation & engagement	Autonomy	Mortality	Self-sufficiency
	Psychological well-being			Pain and other symptoms	
	Social participation/relationships				
	Resilience				
	Enjoyment of life				
Experience	Person-centeredness		Burden of medication	Compassionate care	
	Continuity of care		Burden of informal caregiving	Timely access to care	
				Preferred place of death	
				Burden of informal caregiving	
Costs	Total health- and social care costs	Ambulatory care sensitive hospital admissions	Living at home		Justice contacts
		Hospital re-admissions	Falls leading to ER or hospital admissions		

*ER* Emergency room

Table 2 also shows supplementary sets of outcome measures for each of the four types of integrated care programmes. In addition to the core set and the programme-type specific sets of outcomes, our approach provides researchers with flexibility to use other outcomes, but these outcomes are not included in the MCDA, because their relative importance is not elicited.

The outcomes in Table 2 were defined at a conceptual level and the leaders of the MCDA-work package, provided recommendations to the other SELFIE partners for instruments or indicators that best operationalise these concepts. Where possible we have chosen (domains) of validated instruments (See Additional file 1). When translated versions of the instruments were unavailable, the SELFIE partners have translated them into their own language, using an identical translation protocol with forward and backward translations by native speakers. The chosen instruments were combined into a SELFIE-questionnaire, which varied depending on the type of programme being evaluated.

### Measuring performance (step 3)

The third step in our MCDA is to measure the performance of the 17 integrated care programmes on the selected outcome measures. Therefore, an empirical evaluation was designed in close collaboration with the providers and managers of each programme. Table 3 describes the participants included in the intervention and comparator groups of the 17 programmes. More details on the selection and inclusion of participants per programme can be found in Additional file 2. We adhered to the national regulations regarding medical ethics approvals and waivers and all participants provide written informed consent before participation. As Table 3 shows, the study designs differ across the programmes but most of them are quasi-experimental designs or natural experiments [32]. Like experimental designs, the purpose is to investigate the causal relationship between the outcomes and the exposure (i.e., integrated care), but there is no randomisation of individuals to the intervention and comparator groups. One of the main risks of non-randomised designs is confounding by indication, which precludes unbiased causal inference. To address this, studies will make use of (propensity score) matching or apply a regression discontinuity design [33] to increase the comparability of the comparator group to the intervention group. Furthermore, studies apply regression adjustment and inverse probability weighting to adjust for observed confounding [34], or difference-in-differences analysis [35] to address unobserved confounding. Combinations of these adjustments for confounding are also reported in the literature [36]. In SELFIE, most evaluations use a combination of retrospective data (retrieved from existing databases) with prospective data (collected by questionnaire) with multiple measurement-points per individual in both the intervention and comparator group.

**Table 3:** Study design of the 17 integrated care programmes for individuals with multi-morbidity

Country/Programme	Study design	Intervention group	Comparator group	Data collection/Sample size
Austria				
Health Network Tennengau (HNT)	Cross-sectional and retrospective quasi-experimental; PSM	Residents of Tennengau region in Salzburg receiving integrated care services from HNT, a network of social and health service providers and voluntary organisations	Residents of similar region in Salzburg, insured by the same regional health insurance fund as the intervention group, not treated by HNT	(1) Population-level claims data of all residents of Tennengau and comparator region; n~ 37,000 per group (2) SELFIE-questionnaire administered once to clients of HNT with multiple chronic conditions and a sample of similar individuals of the comparator region; n~ 155 per group; data from (2) are linked to claims data
Sociomedical Centre Liebenau (SMC)	Cross-sectional and retrospective quasi-experimental; PSM	Drug users receiving services by SMC, insured at the regional health insurance fund of the state of Styria	Drug users treated by other facilities offering usual care, insured at the regional health insurance fund of the state of Styria	(1) SELFIE-questionnaire administered once in intervention and comparator group; n~ 70 in intervention group and n~ 150 in comparator group; data from (1) are linked to claims data (2) Individual-level claims data; n~ 70 per in intervention group and n~ 150 in comparator group
Croatia				
GeroS	Prospective quasi-experimental; PSM	Geriatric patients in 2 homes for elderly that provide integrated care using specific modules to monitor and evaluate health needs and functional ability	Geriatric patients in 2 different homes for elderly that have not implemented the GeroS modules	(1) SELFIE-questionnaire administered at baseline and after 6 and 12 months; n~ 200 per group (2) Data from (1) linked to data from health insurers, GPs, and social care information systems; n~ 200 per group
Mobile Multi-disciplinary Specialist Palliative Care Team (MMSPCT)	Prospective quasi-experimental; PSM	Palliative care patients from 3 counties that implemented the MMSPCT	Palliative care patients from 3 different counties that have not implemented the MMSPCT	(1) SELFIE-questionnaire administered at 1st home visit and after 1 and 3 months; n~ 200 per group (2) Data from (1) linked to data from health insurers, GPs, and social care information systems; n~ 200 per group
Germany				
Casaplus	(A) Cross-sectional and retrospective quasi-experimental; difference in difference analyses (B) Prospective before-after study	(A) People ≥55 yrs. with multiple chronic conditions and a high risk of hospitalization, insured by Viactiv BKK, receiving case management incl. a mandatory risk assessment, individual education, a 24/7 crisis service (B) People newly enrolled in the Casaplus programme described above	(A) People ≥55 years with high hospitalization risk insured by AOK receiving usual care (B) No comparator group	(A) Claims data of all individuals enrolled in Casaplus in the years 2013–2018; n~ 1500 in the intervention group and max. 500,000 in comparator group (B) SELFIE-questionnaire administered at baseline and after 12 months; n~ 200 per group
Gesundes Kinzigtal (GK)	(A) Retrospective quasi-experimental; PSM (B) Cross-sectional	(A) Residents of the Kinzigtal region insured by LKK/AOK enrolled in GK population health management (B) Enrollees of GK that visit GP or specialist between Sept and Dec 2017	(A) Residents of the Kinzigtal region insured by LKK/AOK not enrolled in GK (B) Residents of Kinzigtal not enrolled in GK that visit GP or specialist between Sept and Dec 2017	(A) 2005–2016 claims data of all LKK/AOK insured enrolled in GK and ~ 20,000 LKK insured not enrolled (B) SELFIE-questionnaire administered once in both groups; n~ 300 in intervention and n~ 2100 in comparator group
Hungary				
Onko Network	(A) Prospective quasi-experimental study; multi-variate regression (B) Comparison of cohort before and cohort after Onkonetwork; multivariate regression	(A) Target population newly admitted to the hospitals that implemented OnkoNetwork, i.e., individual path management (B) Cohort of individuals suspected of solid tumour in year after implementing OnkoNetwork	(A) Target population newly admitted to a hospital that had not implemented OnkoNetwork (B) Cohort of individuals suspected of solid tumour in year before implementing OnkoNetwork	(A) SELFIE questionnaire administered at first suspect of cancer, at time of the Tumour Board meeting and 6 months after start treatment; data from electronic health records; n~ 300 in each group (B) Data from medical systems before OnkoNetwork (sept 2014-aug 2015) and after OnkoNetwork (Dec 2015-Nov 2016); n~ 3600 in year before and n~ 3600 in year after
Palliative Care Consult Service (PCCS)	(A) Prospective quasi-experimental study; regression + propensity score weighting (B) Retrospective quasi-experimental study; regression + propensity score weighting	(A) Cancer patients with low performance status score for whom the PCCS is newly requested (B) Metastatic cancer patients for whom the PCCS was requested	(A) Comparable cancer patients from the same hospital for whom the PCCS is not requested (some physicians refer to the PCCS, others don’t) (B) Comparable metastatic cancer patients from the same hospital for whom the PCCS is not requested	(A) SELFIE questionnaire administered at hospital admission, hospital discharge and 1 month after discharge; data from electronic health records; n~ 80–100 in intervention and 200–250 in comparator group (B) Hospital administrative and claims data from Jan 2014-Dec 2016; n~ 500–600 in intervention and 1500–2000 in comparator group
Netherlands				
Proactive Primary Care Approach for Frail Elderly (U-PROFIT)	(A) Prospective Regression Discontinuity design (B) Re-analysis of cluster RCT extending the follow-up	(A) Frail elderly ≥75 living at home, identified by screening with U-PRIM who participate in U-PROFIT care programme (B) Frail elderly ≥60 in the U-PRIM or the U-PRIM+U-PROFIT group of a cluster RCT	(A) Frail elderly just below 75 from the same GP practices living at home, identified by screening with U-PRIM who do not participate in U-PROFIT (B) Frail elderly ≥60 in control group of cluster RCT not receiving U-PRIM or U-PROFIT	(A) (1) A questionnaire (with additional items from the SELFIE questionnaire) administered at baseline and after 12 months in each group; n = 480 in intervention and 130 in comparator group (2) Data from (1) are linked to claims data (B) Re-analysis of cluster RCT extending the follow-up for the claims data (from 2000 to 2016 instead of 2013); n = 790 in U-PRIM only, n = 1446 in U-PRIM & U-CARE, and n = 856 in the comparator group.
Care Chain Frail Elderly (CCFE)	Prospective quasi-experimental, PSM	Frail elderly living at home with complex care needs and loss of control, from 3 primary care groups participating in a bundled care programme for frail elderly	Similar frail elderly from same region, receiving usual care from GPs of 1 the 3 primary care groups that not implemented the programme	(1) SELFIE-questionnaire administered to elderly at baseline and after 6 and 12 months in each group; n~ 200 per group (2) Data from (1) are linked to claims data, data from electronic medical records and GP information systems (3) CarerQol administered to related informal caregivers at baseline and after 6 and 12 months; n~ 100 per group
Better Together in Amsterdam North (BSiN)	Prospective quasi-experimental, PSM	Individuals with limited self-sufficiency in multiple life domains referred for participation in BSiN programme	Individuals with limited self-sufficiency identified in the ‘Amsterdam Health Monitor’	(1) A questionnaire (with additional items from the SELFIE questionnaire) administered at baseline and after 6 and 12 months in each group; n~ 70 per group (2) Data from (1) are linked to claims data from same period
Norway				
Learning Networks	Prospective quasi-experimental, PSM	Frail elderly referred to home care services or a short-term stay in a nursing home who are newly enrolled in a programme for whole, coordinated and safe care pathways offered by 11 municipalities	A similar group of frail elderly from similar municipalities who do not offer such a care pathway programme	(1) SELFIE questionnaire at 2 fixed time periods, 6 months apart; n = 300 per group (2) Municipality-level registry information on centrality, staffing, economics etc. over the years 2017–2018
Medically Assisted Rehabilitation Bergen	Prospective and retrospective quasi-experimental, PSM	People with opioid addiction participating in a programme integrating health and social care services of specialists and the municipalities in Bergen	People with opioid addiction participating in a conventional care programme in Oslo, Stavanger and Trondheim	(1) SELFIE questionnaire in Bergen at 2 fixed time periods, 12 months apart (2) Data from Status report (SERAF) over 2016 and 2017; n = 300 in intervention group and n = 300 in comparator group (3) National registry data over 2016 and 2017; n = 300 in intervention group and n = 300 in comparator group
Spain				
Barcelona-Esquerra (AISBE)	(A) Retrospective quasi-experimental population-based evaluation, PSM (B) Cross-sectional programme-component evaluation	(A) Residents served by the Barcelona-Esquerra healthcare provider organizations that offer integrated care services for chronic patients across healthcare tiers. (B) Patients admitted to the hospital at home/early discharge programme offered by Hospital Clinic	(A) Residents of the entire region and residents served by other provider organisations in the same region of Barcelona-Esquerra (B) Comparable group of patients from a comparable hospital (Hospital Sagrat Cor) that does not offer hospital at home/early discharge	(A) Data from Catalan Health Surveillance system of 540,000 residents in AISBE over the years 2011 to 2017 and a similar number in the comparator group. (B) (1) SELFIE questionnaire administered at 1 month and 6 months post-discharge; n = 200 per group (2) Data from (1) are linked to data from electronic medical records of hospitals and primary care providers
Badalona Serveis Assistencials (BSA)	Prospective and retrospective quasi-experimental, PSM	Individuals living in Badalona who participate in BSA’s integrated care programme for frail elderly that includes: (i) Early Discharge support; (ii) Long-term home-based support services and (iii) Residential care	For each of the three intervention groups, a corresponding control group was selected among individuals living in Badalona but attended by providers or living in residencies not included in the BSA program	(1) For service (i): SELFIE questionnaire administered at start of service and 3 months thereafter; n = 50 per group (2) For service (ii) and (iii): SELFIE questionnaire administered once; n = 50 per group (service ii) and n = 100 per group (service iii) (3) Data from (1) and (2) are linked to data from electronic medical records of hospitals and primary care providers (4) For the evaluation of the BSA’s entire integrated frail elderly care approach: registry data from the Catalan Health Surveillance System over the years 2011–2017; n = 2000 per group
UK				
Salford Integrated Care Programme (SICP)/Salford Together	(A) Retrospective quasi-experimental population-based evaluation; difference-in-differences analyses (using matching), exploiting gradual roll-out and geographical limits, and examining differential effect by multi-morbidity status. (B) Retrospective quasi-experimental programme-component evaluation	(A) Individuals 65+ with long-term conditions that are eligible for the following 3 services by 1 clinical commissioning group, i.e., case management services and self-management. (B) Individuals 65+ receiving case-management, community groups, a centralised telephone hub to help with navigating	(A) Entire population of 65+ in England and populations of 65+ from other geographical regions (i.e., other clinical commissioning groups not offering a similar integrated care programme) and other time periods (B) Salford population 65+ with similar multi-morbidity not receiving case management	(A) Routinely collected population-level English NHS data (Hospital Episode Statistics and GP Patient Survey) over the years 2011–2016; n~ 35,000 65+ in Salford and n~ 9.3 million 65+ in England as a whole (B) Re-analysis of data from CLASSIC cohort study over the years 2014–2015 including the core outcome-concepts of SELFIE; n~ 4000 65+ in CLASSIC; n~ 35,000 65+ Salford population
*South Somerset Symphony Programme (SSSP)*	(A) Retrospective quasi-experimental population-based evaluation; difference-in-differences analyses (using matching if necessary), exploiting gradual roll-out and geographical limits, and examining differential effect by multi-morbidity status. (B) Retrospective quasi-experimental programme-components evaluation	(A) Population of the Clinical Commissioning Group that offers the SSSP including complex care hubs of GPs in the hospital and co-location of health coaches in all GP practices (B) i) Individuals using the complex care hubs ii) Individuals in GP practices incorporating health coaches (enhanced primary care) as this was gradually rolled out in three waves	(A) Entire population of England and other geographical regions and other time periods (B) (i) Propensity matched persons within South Somerset not using the complex care hubs (ii) Practices act as controls until they roll-out the intervention	(A) Routinely collected population-level English NHS data (Hospital Episode Statistics and GP Patient Survey) over the years 2011–2016; n~ 115,000 (1500 with 3 or more selected chronic conditions that the programme initially focused on) in South Somerset and n~ 54.8 million (0,5 million with 3 or more conditions) in England as a whole (B) Routinely collected population-level English NHS data (Hospital Episode Statistics and GP Patient Survey); (i) n~ 750 in intervention group and n~ 1500 in comparator group; (ii) 19 GP practices joining intervention in three waves

*PSM* Propensity Score Matching, *BKK* BetriebsKrankenKasse, *AOK* Algemeine OrtskrankenKasse, *PHM* population health management

### Weighting the criteria (step 4)

In the fourth step of our MCDA, a Discrete Choice Experiment (DCE) [37] is conducted in each country in the SELFIE project to obtain the weights (or relative importance) that the 5P stakeholder groups assign to the core set of outcomes. In addition, Swing Weighting [38] is used to elicit weights for both the programme-type specific sets of criteria and the core set. These two preference elicitation methods were chosen because they force stakeholders to trade criteria off against one-another, as opposed to merely rating a single criterion [39]. Moreover, they take account of the entire range of potential performance of integrated care programmes, which is of particular importance for the applicability of the weights to future MCDA evaluations. We choose to apply two different weighting methods because DCE, although theoretically very well-founded [40], allows only for a limited set of criteria due to cognitive burden. For this reason, and due to the aforementioned benefits, swing weighting was applied for the full range of outcome criteria.

**Fig. 2:**
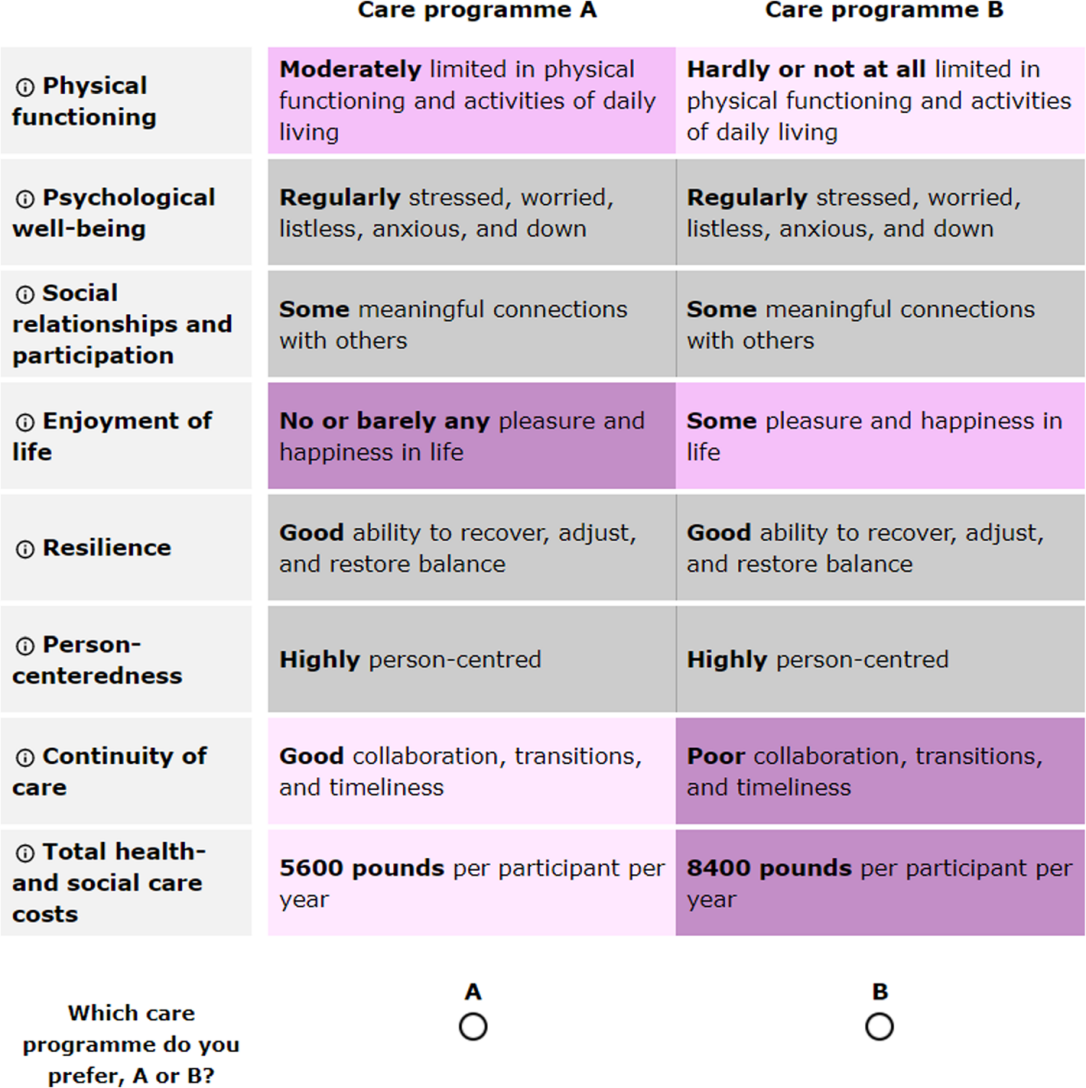
Example of a DCE question in the UK

In the DCE, choice sets with two different integrated care programmes per choice are presented to respondents and they are asked which programme they prefer. The description of the integrated care programmes systematically differs in terms of their performance on the core set of outcome criteria. Each outcome criterion has three levels, generally reflecting a poor, average and good performance on that outcome, framed in general conceptual terms (See Additional file 3). All outcomes and levels were identical between the SELFIE countries, except for costs. The three levels of the costs were based on country-specific estimates of the mean total health and social care costs for people with multi-morbidity in 2017 (middle level) and increased and decreased by 20% to obtain the poor and good performance level. The costs were expressed in the national currency. A D-efficient DCE design [41] with priors from the literature was created with 10 different sub-designs and 18 DCE choice-sets per sub-design; at the outset the same DCE design was used in each questionnaire (8 countries X 5 P stakeholder groups = 40 questionnaires). Each respondent is asked to complete a randomly chosen sub-design with 18 choice-sets. To reduce the overall complexity of the choice tasks and improve response efficiency there is level-overlap for 4 and 5 of the 8 outcomes in one choice set. To optimise the D-efficient DCE design, the priors were updated after the first circa 50 respondents in a stakeholder group within a particular country completed the questions (i.e., 40 updates). An example of a DCE question is shown in Fig. 2. The weights for each criterion-level are statistically estimated from the likelihood that one scenario, with specific criteria performance, is preferred over another. The relative weights of best levels of each outcome criterion are used in the calculation of the overall value score in the next step of the MCDA.

In Swing Weighting, and specifically in the applied Simple Multi-Attribute Rating Techniques Exploiting Ranks (SMARTER) method [27, 42], respondents get a description of an integrated care programme that has the worst possible level of performance on all outcome criteria. They are asked which criterion they would select first to improve (i.e., to swing) from the worst to the best level. After the chosen criterion is removed from the entire set of criteria, they are asked which criterion they would select second. This is continued on until all criteria are ranked. The resulting rank order is then turned into weights, for example, by using the rank ordered centroid method [43]. The tables in Additional file 4 include a description of the worst and the best level in Swing weighting; for the outcome criteria that are also included in the DCE the wording of these levels is the same as for the poor and the good level in the DCE.

An online weight-elicitation questionnaire was created that contained a brief introduction to integrated care in the European SELFIE project, a general explanation of the type of questions and the perspective from which the questions should be completed (i.e. 1 of the 5 Ps), a detailed instruction on how to complete the DCE and Swing Weighting questions plus examples to practice before answering the real questions, three blocks of 6 DCE questions with some demographic questions in between, the Swing Weighting question, and a multiple-choice question on the level of difficulty of the questionnaire. Definitions of the outcome criteria were provided and when respondents navigated over an outcome-heading in the DCE and the Swing Weighting, the definition of that outcome would appear. As can been seen in Fig. 2 colour coding was used in the DCE questions, and outcomes that had the same level in the two integrated care programmes were presented in grey. Colour coding was also used in the Swing Weighting where the arrows in between the worst and best level of an outcome criterion changed from red to green. The entire weight-elicitation questionnaire was pilot tested in patients and elderly. To translate the English questionnaire into the various languages, each country used the same translation protocol including forward and backward translations by native-language speakers with an excellent level of English. The number of Swing Weighting questions differs per country depending on which types of programmes are being evaluated in a particular country. The SELFIE partners translated the weight-elicitation questionnaire into their own language, using the same translation protocol as for the performance-score questionnaire (described in step 3). Each country in the SELFIE project had a target of recruiting a minimum of 150 respondents from each of the 5Ps, the sample size required to detect significant main effects in the DCE [44]. Patients, Partners, and Professionals are mostly recruited via professional panel organisations, or organisations representing patients, informal carers, or professional care providers. The strategies to recruit Payers and Policy makers include snowballing, starting with the identification of organisations of payers and policy makers in a country, reaching out to them via one or more individuals known to the SELFIE consortium, and asking them to recruit other respondents within their organisations.

### Creating an overall value score (step 5)

In a multi-attribute value-based method of MCDA the performance scores of the integrated and usual care programmes (derived in step 3) and the weights of the outcomes (derived in step 4) are combined into an overall value for the integrated care programme and its comparator, using a ‘weighted sum approach’ [26, 39]. This fifth step is illustrated with a hypothetical example in Table 4, which shows the (standardised) performance scores of two hypothetical care programmes (e.g., integrated vs. usual care) on the core set of outcomes (i.e., criteria), the weights of these criteria from the viewpoint of two different stakeholder groups (P1 and P2), and the weighted aggregation. The performance scores are standardised to remove the impact of differences in their scales. In this example the aggregated score for ‘enjoyment of life’ is calculated by multiplying the criteria weight of stakeholder group 1 (0.30) or stakeholder group 2 (0.15) with the standardised performance (0.80 for the integrated care programme and 0.60 for the comparator). When these weighted performance scores are summed across all criteria the overall value of a programme is obtained. In this example the first stakeholder group prefers the integrated care programme over the comparator because it performs better on five of the eight outcomes that are important to them. The second stakeholder group prefers the comparator, which performs better on social participation, physical functioning, and costs; the latter two outcomes were also considered more important by this stakeholder group than by the first stakeholder group.

**Table 4:** Calculating overall value scores

	Range performance score	Performance		Standardised performance^a^		Weights		Weighted aggregation			
								Integrated care		Comparator	
	worst-best	Integrated care	Comparator	Integrated care	Comparator	P1	P2	P1	P2	P1	P2
Health & well-being											
Physical functioning	0–100	60	70	0.65	0.76	0.100	0.250	0.065	0.163	0.076	0.190
Psychological well-being	0–100	70	50	0.81	0.58	0.150	0.100	0.122	0.081	0.087	0.058
Social participation & relationships	0–4	3	4	0.60	0.80	0.125	0.100	0.075	0.060	0.100	0.080
Resilience	1–5	2	4	0.45	0.89	0.050	0.100	0.022	0.045	0.045	0.089
Enjoyment of life	0–4	4	3	0.80	0.60	0.300	0.150	0.240	0.120	0.180	0.090
Experience											
Person-centeredness	1–4	4	3	0.80	0.60	0.100	0.050	0.080	0.040	0.060	0.030
Continuity of care	1–5	5	3	0.86	0.51	0.125	0.050	0.107	0.043	0.064	0.026
Costs											
Total health and social care costs	8500–5500	8000	6000	0.20	0.40	0.050	0.200	0.010	0.040	0.020	0.080
Overall value score								**0.722**	0.592	0.632	**0.643**

Performance: hypothetical average performance values, Weights: hypothetical weights obtained in DCE for stakeholder group 1 (P1) and 2 (P2), weighted aggregation: aggregation of standardised performance measures using weights for each stakeholder group

^a^Performance scores are standardised with the following formula: ${S}_{aj}=\frac{x_{aj}}{{\left({x}_{aj}^2+\kern0.5em {x}_{bj}^2\right)}^{1/2}}$, where *x* = performance score on the natural scale, *a* = integrated care, *b* = comparator, *j* = criteria j

### Sensitivity analyses and interpretation of results (step 6, 7)

In the sixth step, we address the uncertainty in the MCDA results by performing a series of deterministic sensitivity analyses. These include, for example, the exclusion of certain criteria (e.g., the most dominating), the use of weights obtained by Swing Weighting rather than DCE, and the pooling of criteria-weights from different stakeholder groups. Furthermore, we model the parameter uncertainty in the performance scores and the criteria-weights simultaneously in a probabilistic sensitivity analysis using Monte-Carlo simulation [45, 46]. In this analysis, the joint uncertainty can be presented graphically on an acceptability curve where the vertical axis shows the probability of an integrated care programme to be accepted as the preferred alternative against the comparator and the horizontal axis shows different thresholds of maximum budget available to be allocated to either intervention or comparator, for the treatment of a given population-size. The curve shows, for a range of available budgets, the likelihood that the integrated care programme is the preferred alternative (i.e., has the highest overall value score) while the budget-impact stays below a budget-threshold. This new way of representing uncertainty in MCDA may be called a Conditional Multi-attribute Acceptability Curve (CMAC). Although the CMAC was inspired by the cost-effectiveness acceptability curve (CEAC) [47], it differs because the probability of the evaluated intervention to be cost-effective is based on various outcomes relevant for decision-making beyond the quality-adjusted life year (QALY), and the budget available for the evaluated intervention is more appealing and adaptable to decision-makers at all levels than the monetary value of a QALY.

In the seventh and last step of the MCDA, the findings and their robustness in the sensitivity analyses are interpreted and reflected upon by the researchers together with representatives from the 5Ps. This is done in national workshops in the SELFIE partner countries and in an international workshop. The explication of discrepancies between different perspectives and the impact this had on the relative importance of criteria and the final results of the MCDA is expected to stimulate debate about the reasons underlying the differences in perspectives. Ultimately, the MCDA will support the decisions to be made regarding the reimbursement, continuation, extension, and/or wider implementation of integrated care programmes.

## Discussion

Because resources are scarce, investing in integrated care interventions either displaces other health care interventions or requires additional financial resources from taxes, health insurance premiums, and/or patient co-payments. Therefore, payers and policy makers are keen to ensure that they allocate scarce healthcare resources only to services that have proven added value. In the SELFIE project, we evaluate the added value of integrated care programmes using MCDA, because that offers an evaluation framework in which a broader definition of value can be used, which is highly relevant to the evaluation of integrated care programmes for individuals with multi-morbidity. In the SELFIE project, we broaden the scope of outcomes to evaluate the added value towards the Triple Aim. Moreover, because the outcomes are weighted, an MCDA makes underlying preferences explicit and can be done from multiple perspectives, i.e., in SELFIE from the perspectives of the five stakeholder groups. Designing and updating 40 DCE’s (8 countries × 5 stakeholder groups) is quite unique in this type of research, enabling extensive cross-country and cross-stakeholder group comparisons of the relative weights. We believe that the systematic and explicit trade-off between multiple, sometimes conflicting, outcomes in MCDA’s from different perspectives can improve the transparency, consistency, accountability, credibility and acceptability of policy decisions about integrated care programmes for individuals with multi-morbidity. However, developing a uniform MCDA approach for application in the eight European countries participating in the SELFIE project is associated with many challenges. In this section, we discuss these challenges and the solutions that we choose in SELFIE to address them.

### Common set of outcomes

Considering the variation in target groups and interventions provided in the selected integrated care programmes, one of the first challenges was to define a common set of outcomes to be measured. Given our plan to use a more holistic, person-centred, understanding of added value, we agreed on a minimum data set of eight outcomes that mainly included patient-reported outcomes and experience measures (PROMS and PREMS) that cover the Triple Aim. Besides physical, mental and social well-being from the 1984 WHO definition of health [48], the core set includes resilience and enjoyment of life, two aspects of more positive and active definitions of health, such as health as the ability to adapt presented by Huber et al. [49, 50]. Moreover, it includes two indicators of the experienced care process, i.e., person-centeredness and continuity of care. These outcomes were considered highly important by the persons with multi-morbidity that participated in the focus groups.

We deliberately defined the outcomes at a conceptual level in order to allow for the use of different instruments/indicators to measure a particular outcome because some programmes have already been measuring outcomes with certain instruments/indicators for years. The advantage of having longitudinal data with the previously used instruments was thought to offset the disadvantage of not having exactly the same instruments as included in the SELFIE-questionnaire. However, this creates the challenge of ensuring that these instruments are conceptually similar enough to justify the application of the same weight in the MCDA. This requires a careful content-mapping of the instruments to the outcomes as defined in SELFIE.

### Including costs in MCDA

Related to the choice of criteria in an MCDA, is the debate about whether or not to include costs as a criterion in the MCDA analysis. Those who argue against including costs in MCDA, argue that MCDA creates a new composite score of benefit and that the main question to be answered is what the opportunity costs are of one unit of additional benefit on that composite score [51, 52]. In other words, how much money can be spent at maximum for one unit of this composite score? Those who are in favour of including costs, however, argue that each MCDA will result in a different composite score, dependent on what criteria are included. This seems to make it difficult to determine a threshold for a unit of improvement [39]. They argue that by including costs in the weight-elicitation process respondents explicitly trade costs off against the other criteria, making their relative contribution throughout the entire decision-making process explicit. This is seen as being equivalent to estimating willingness-to-pay values for benefits [39]. In SELFIE, costs are included in the MCDA. We acknowledge that by including costs as one of the criteria we do not adequately address the opportunity costs of alternative uses of resources. In most of the 17 programmes the decision context is whether to continue or roll-out piloted integrated care programmes, and the principle decision to invest in integrated care has already been made. Hence, the local question that remains is whether the particular integrated care programme evaluated generates sufficient benefits over the comparator to justify allocation of resources to that particular programme. Benefits of integrated care programmes are commonly expressed in terms of the extent to which the Triple Aims are achieved. Reducing costs is one of these aims, and hence it cannot be seen separately from other outcome criteria.

Having said that, there are several SELFIE-partners (HU, NL, NO, ES, UK) who, in addition to the MCDA, perform a cost-utility analysis of the integrated care programme versus usual care, using the EQ-5D-5L [53] to calculate utilities and QALYs. This will allow for a comparison between the conclusions of both types of evaluations.

### Quasi-experimental study designs

Generating scientifically rigorous evidence is particularly challenging for complex interventions that involve organisational or system-level changes, like the 17 selected programmes [54]. In contrast to many previous evaluations of integrated care programmes, in SELFIE the outcomes are usually measured at least twice over time, and/or data that are extracted from existing sources cover multiple years in both the intervention and the comparator group. For most programmes it was possible to identify comparable control groups. However, randomisation of individual patients was considered inappropriate because of the contamination into ‘usual care’ that results from the interventions directed at the professionals and other staff, entire organisations or systems in an integrated-care programme. Also, the intervention might already be in a developed stage and widespread use, raising ethical concerns about randomisation and withholding treatment in the control group. Even randomisation of practices, organisations or regions in cluster-RCTs is often impossible, because there might not be enough suitable organisations to be randomised. Hence, most evaluations use quasi-experimental study designs in which they need to apply appropriate statistical techniques to increase the comparability of the intervention and the comparator group for causal inference. One-to-one propensity score matching is often not an option because the sample from which statistical twins may be drawn is too small. Therefore, several evaluations apply inverse probability weighting in which the propensity scores are used to weigh the outcomes estimated by repeated measurements regression equations. In contrast to one-to-one matching, no cases have to be excluded from the comparator group in this method. To assess the increased comparability between the intervention and comparator group after application of inverse probability weighting, standardised differences in baseline individual- and disease-characteristics before and after weighting are reported.

### Evaluating population health management programmes

The study designs that were most heavily debated in the SELFIE consortium were those to evaluate the population health management programmes [55]. One of the reasons was that these programmes ‘in principle’ target the entire population in a region, making it impossible to form a usual care or comparator within the same region. Another reason was that these programmes may include a mixture of very different interventions ranging from health promotion and prevention to rehabilitation and end-of-life support. Each of these interventions may target a different segment of the population and there may be a segment of the population that has never been (directly) exposed to a particular intervention at all. After extensive debate in the SELFIE consortium, we were able to define an appropriate comparator group for each of the selected population-health management programmes. These groups either consist of the entire population living in a different geographical region, people being insured by a different health insurer within the same region that does not offer integrated care, people receiving care from different providers not offering integrated care, or national-level population data. However, if a population health management programme targets people insured by particular insurers, there may be some people from other insurers (i.e., the comparator group) who also benefit from the new population health management approach adopted by the providers (i.e., a spill-over effect at the professional-level). Furthermore, unlike the other programmes that apply prospective evaluations in which PROMS and PREMS are repeatedly measured in the same individuals, some population health management programmes could only conduct cross-sectional measurements of the PROMS and PREMS, mostly for feasibility reasons. Defining the sample for measurement was difficult because subgroups of the population are exposed to different interventions. Therefore, some of the evaluations in SELFIE have added programme-component evaluations besides the evaluation of the entire population health management programme. This limitation is compensated by the availability of a wide range of routinely collected population-level health surveillance data, claims data and structure and process data over many years in both intervention and control group, which allows for difference-in-differences analyses on the entire population. The latter analyses are done in addition to the MCDA.

### Weight-elicitation

Although trading-off multiple outcomes is one of the strengths of MCDA, the large number of outcomes for which we had to obtain weights was a challenge. We considered DCE’s with a partial profile design in which each choice set includes only a subset of the attributes (i.e., outcomes). However, the assumption underlying partial profiling, namely that the attributes not shown do not influence the scoring has proven to be invalid; respondents do make assumptions about the attributes not shown [56]. In countries where two different types of integrated care programmes are evaluated, and thus two sets of programme-type specific outcomes are present, the respondents may have to value up to 18 outcomes, which is only feasible with Swing Weighting. In the end, our approach results in two different sets of relative weights, one for the core set of outcomes based on DCEs, and one for the core set and the programme-type specific set based on Swing Weighting. These weight sets are not directly comparable. This calls for sensitivity analysis to investigate how sensitive the outcomes are to these differences in methodology.

A major strength of an MCDA is that the weights can be obtained from multiple groups of stakeholders. We decided to obtain weights from representatives of the 5P’s in order to inform decision makers about the extent to which different roles lead to different opinions about the importance of certain outcomes. This raises normative issues such as the question about whose preferences should count most. In the end, it is up to the decision makers to weigh the preferences of different stakeholder groups to make a well-informed final decision.

Including five different stakeholder groups also creates the challenge of recruiting a high number of respondents. Finding the required number of 150 respondents for the DCE among the Payers and the Policy makers may be more difficult than recruiting 150 Patients, Partners, and Professionals. This is especially the case in counties with relatively few people working in health- and/or social care policy making/advising (e.g., the Eastern European Countries), smaller countries, or countries where there is a single payer (i.e., a National Health Service). Fortunately, we need less respondents per stakeholder group for the Swing Weighting because in that method less parameters have to be estimated.

## Conclusion

In conclusion, we described a methodologically innovative mixed-methods approach to perform MCDAs of 17 integrated care programmes for individuals with multi-morbidity that we apply in 8 countries. This approach includes qualitative research to understand the details and decision context of the programmes and quantitative research to measure performance on and weights of a core set of outcomes to be used across all programmes and four sets of programme-type specific outcomes. This offers unique opportunities to investigate how cross-country, cross-stakeholder and cross-method differences in weights affect the MCDA outcomes. The SELFIE MCDA framework can be used to improve the transparency, consistency, accountability, credibility and acceptability of the decision making about the implementation of integrated care for people with multi-morbidity. The framework can also be used in future evaluation studies across Europe and beyond.

## Additional files

Additional file 1: **Table S1.** Instruments recommended to measure the core set of outcomes. (DOCX 14 kb)

Additional file 2: **Table S2.** Selection of patients in the intervention and control groups. (DOCX 28 kb)

Additional file 3: **Table S3.** Definition of outcome criteria (attributes) and levels in the DCE. (DOCX 14 kb)

Additional file 4: **Table S4-S7.** Supplementary outcome criteria and their worst and best levels in Swing Weighting. (DOCX 19 kb)

## Abbreviations

CEAC: Cost Effectiveness Acceptability Curve
CMAC: Conditional Multi-Attribute Acceptability Curve
DCE: Discrete Choice Experiment
ES: Spain
HbA1c: Hemoglobine A1c
HU: Hungary
ICT: Information and Communication Technology
MCDA: Multi-Criteria Decision Analysis
NL: Netherlands
NO: Norway
PREMS: Patient-Reported Experience Measures
PROMS: Patient-Reported Outcome Measures
QALY: Quality Adjusted Life Year
RCT: Randomised Controlled Trial
SELFIE: Sustainable intEgrated chronic care modeLs for multi-morbidity: delivery, FInancing, and performance
SMARTER: Simple Multi-Attribute Rating Techniques Exploiting Ranks
UK: United Kingdom
