# Supplementary material for: Strengthening the evidence-base of integrated care for people with multi-morbidity in Europe using Multi-Criteria Decision Analysis (MCDA)
Source: BMC Health Serv Res. 2018 Jul 24;18:576. doi: 10.1186/s12913-018-3367-4 (PMC6057041; doi:10.1186/s12913-018-3367-4)
Supplement: Supplementary file 1 — Table S1. Instruments recommended to measure the core set of outcomes. (DOCX 14 kb) [file 12913_2018_3367_MOESM1_ESM.docx]

Table S1. Instruments recommended to measure the core set of outcomes.

|  | **Outcome** | **Instrument** |
| --- | --- | --- |
| **Health & well-being** | Physical functioning | SF-36 (physical functioning domain) or Katz-15 for ADL |
|  | Psychological well-being | MHI-5 |
|  | Social participation/relationships | IPA (social life and relationships domain) |
|  | Resilience | BRS |
|  | Enjoyment of life | ICECAP-O (item on enjoyment and pleasure) or Q-LES-Q (item on life satisfaction) |
| **Experience** | Person-centeredness | P3CEQ (experience of person-centered care domain) |
|  | Continuity of care | NCQ (Team and cross boundary continuity domain) + CPCQ (item on waiting for appointment/treatment) |
| **Costs** | Total health- and social care costs | Based on iMTA Medical Consumption Questionnaire |

SF-36: Short Form 36, ADL: Activities of Daily Living, ICECAP-O: Investigating Choice Experiments for the preferences of Older people CAPability measure , Q-LES-Q: Quality of Life, Enjoyment and Satisfaction Questionnaire, IPA: Impact on Participation and Autonomy, BRS: Brief Resilience Scale, P3CEQ: The Person-centered Coordinated Care Experience Questionnaire, NCQ: Nijmegen Continuity Questionnaire, CPCQ: Client Perceptions of Coordination Questionnaire, iMTA: Institute for Medical Technology Assessment
