# Supplementary material for: Strengthening the evidence-base of integrated care for people with multi-morbidity in Europe using Multi-Criteria Decision Analysis (MCDA)
Source: BMC Health Serv Res. 2018 Jul 24;18:576. doi: 10.1186/s12913-018-3367-4 (PMC6057041; doi:10.1186/s12913-018-3367-4)
Supplement: Supplementary file 2 — Table S2. Selection of patients in the intervention and control groups. (DOCX 28 kb) [file 12913_2018_3367_MOESM2_ESM.docx]

Table S2. Selection of patients in the intervention and control groups

|  | **Study Design** | **Intervention group** | **Comparator Group** |
| --- | --- | --- | --- |
| **Austria** | | | |
| *Health Network Tennengau (HNT)* | Cross-sectional and retrospective quasi-experimental; PSM | Patients have to fulfil the following inclusion criteria: 1) receiving integrated care services from HNT, 2) residents of Tennengau region in Salzburg, 3) having multiple chronic conditions, 4) aged 18 and over, 5) insured at the regional health insurance fund of the state of Salzburg.  Patients are identified by the GPs and senior citizen counsellors involved in the HNT. | Patients have to fulfil the following inclusion criteria: 1) NOT receiving integrated care services from HNT,  2) residents of region in Salzburg similar to Tennengau region, 3) having multiple chronic conditions, 4) aged 18 and over, 5) insured at the regional health insurance fund of the state of Salzburg.  Patients are identified by GPs in the regions and by nurses in the course of a separate routine survey. |
| *Sociomedical Centre Liebenau (SMC)* | Cross-sectional and retrospective quasi-experimental; PSM | Drug users 1) receiving substitution therapy from SMC, 2) aged 18 or over, 3) insured at the regional health insurance fund of the state of Styria  Patients are identified by the GP and/or the social worker working in the SMC. | Drug users 1) receiving substitution therapy by other facilities offering usual care in Styria, 2) aged 18 or over, 3) insured at the regional health insurance fund of the state of Styria  Patients are identified by GP offering substitutions therapy in Styria or other facilities (e.g. drug counselling centre). |
| **Croatia** | | | |
| *GeroS* | Prospective quasi-experimental; PSM | Geriatric patients in 2 homes for elderly that provide integrated care using specific modules to monitor and evaluate health needs and functional ability.  Patients have to fulfil the following inclusion criteria: 1) geriatric patients with multi-morbidity who are 65 years or older, 2) a life expectancy of more than 12 months. Patients who are not able to give answers on questionnaires (have a cognitive condition or are unresponsive or nonverbal), and patients unlikely to survive more than 12 months based on their clinicians’ evaluation were excluded. | Geriatric patients in 2 different homes for elderly that have not implemented the GeroS modules.  Patients have to fulfil the following inclusion criteria: geriatric patients with multi-morbidity who are 65 years or older), 2) a life expectancy of more than 12 months. Patients who are not able to give answers on questionnaires (have a cognitive condition or are unresponsive or nonverbal), and patients unlikely to survive more than 12 months based on their clinicians’ evaluation were excluded. |
| *Mobile Multi-disciplinary Specialist Palliative Care Team (MMSPCT)* | Prospective quasi-experimental; PSM | Palliative care patients from 3 counties that implemented the MMSPCT.  Patients were considered for enrolment by the MMSPCT if they were palliative care patients (according to the Supportive and Palliative Care Indicators Tool-SPICTTM and ICD-10: Z51.5), 18 years or older, and had a life expectancy between 1 and 6 months. Patients and/or families who refuse further care by the MMSPCT, who are not able to give answers on questionnaires (have a cognitive condition or are unresponsive or nonverbal), and patients unlikely to survive more than 1 month based on their clinicians’ judgments were excluded. | Palliative care patients from 3 different counties that have not implemented the MMSPCT.  Patients were considered for enrolment by their primary care physicians if they were palliative care patients (according to the Supportive and Palliative Care Indicators Tool-SPICTTM and ICD-10: Z51.5), 18 years or older, and had a life expectancy between 1 and 6 months. Patients and/or families who refuse the usual care, who are not able to give answers on questionnaires (have a cognitive condition or are unresponsive or nonverbal), and patients unlikely to survive more than 1 month based on their clinicians’ judgments were excluded. |
| **Germany** | | | |
| *Casaplus* | (A) Cross-sectional and retrospective quasi-experimental; difference in difference analyses  (B) Prospective before-after study | (A) People ≥55 yrs with multiple chronic conditions and a high risk of hospitalization, insured by Viactiv BKK, receiving case management incl. a mandatory risk assessment, individual education, a 24/7 crisis service.  A claims data based algorithm is used to identify patients with a high hospitalization risk. These patients were contacted and asked if they would like to participate in the programme.  (B) People newly enrolled in the Casaplus programme described above. They were identified in the same way as in (A). In addition, the case manager asked patients if they would like to participate in the evaluation. | (A) Insured ≥55 yrs with multiple chronic conditions in the Viactiv BKK with a high risk of hospitalization receiving usual care.  A claims data based algorithm is used to identify patients with a similar hospitalization risk.  (B) No comparator group |
| *Gesundes Kinzigtal (GK)* | (A) Retrospective quasi-experimental; PSM  (B) Cross-sectional | (A) Residents of the Kinzigtal region insured by LKK/AOK visiting physicians who are enrolled in GK population health management.  Claims data were used to identify physicians who are enrolled in GK. Patients who were visiting these physicians more than once in a year and not visiting physicians not enrolled in GK were considered as intervention group.  (B) Enrollees of GK that visit GP or specialist between Sept and Dec 2017.  Physician assistants distributed questionnaires to all patients who visited their practice in the period between Sept and Dec 2017. Patients who said yes to the question about whether they were participating in the GK programme were enrolled in the intervention group. | A) Residents of the Kinzigtal region insured by LKK/AOK visiting physicians who are not enrolled in GK.  Claims data were used to identify physicians who are not enrolled in GK. Patients who were visiting these physicians more than once in a year and not visiting physicians enrolled in GK were considered as comparator group.  (B) Residents of Kinzigtal not enrolled in GK that visit GP or specialist between Sept and Dec 2017  Physician assistants distribute questionnaires to patients who visit their practice in the period between Sept and Dec 2017. Patients who said no to the question about whether they were participating in the GK programme were enrolled in the control group. |
| **Hungary** | | | |
| *Onko Network* | (A) Prospective quasi-experimental study; multi-variate regression  (B) Comparison of cohort before and cohort after Onkonetwork; multivariate regression | (A) Target population newly admitted to the hospitals that implemented OnkoNetwork. Patients with a new “C” or “D” ICD code were identified and contacted at hospital admission for their informed consent to study inclusion.  (B) Cohort of individuals suspected of solid tumour in the year after implementing OnkoNetwork. All patients with a new “C” or “D” ICD code (except for haematology codes) were identified in the medical system of the hospital and enrolled into the retrospective analysis. | (A) Target population newly admitted to a county hospital that had not implemented OnkoNetwork. Patients with a new “C” or “D” ICD code were identified and contacted at hospital admission for their informed consent to study inclusion.  (B) Cohort of individuals suspected of solid tumour in the year before implementing OnkoNetwork. All patients with a new “C” or “D” ICD code (except for haematology codes) were identified in the medical system of the hospital and enrolled into the retrospective analysis. |
| *Palliative Care Consult Service (PCCS)* | (A) Prospective quasi-experimental study; regression + propensity score weighting  (B) Retrospective quasi-experimental study; regression + propensity score weighting | (A) Cancer patients (ICD code C00-C97) admitted to selected hospital departments with a Karnofsky Performance Status Scale score ≤50 for whom the PCCS was newly requested. Patients with short stays (up to 3 days) and with admissions for routine chemotherapy or hormone therapy were excluded.  (B) Cancer patients (ICD code in the C00-C97 range) with metastasis (based on TNM status or specific ICD codes) admitted to selected hospital departments for whom the PCCS was requested. Patients with short stays (up to 3 days) and with admissions for routine chemotherapy or hormone therapy were excluded. Patients with 180+ days of survival were also excluded. | (A) Comparable cancer patients from the same hospital departments for whom the PCCS was not requested (some physicians refer to the PCCS, others don’t). The same exclusion criteria apply.  (B) Comparable metastatic cancer patients from the same hospital departments for whom the PCCS was not requested. The same exclusion criteria apply. |
| **Netherlands** | | | |
| *Proactive Primary Care Approach for Frail Elderly (U-PROFIT)* | (A) Prospective Regression Discontinuity design  (B) Re-analysis of cluster RCT extending the follow-up | (A) Frail elderly ≥ 75 living at home, identified by screening with U-PRIM who participate in U-PROFIT care programme.  In step 1 of the screening, the electronic medical records are screened to detect if people meet at least 1 of 3 criteria: 1) polypharmacy (≥ 5 medicines), 2) GP consultation gap of ≥ 3 years and 3) multi-morbidity based on the Frailty Index. If they do they are further screened with the Groningen Frailty Index on which they have to score ≥ 4 to be included in U-PROFIT.  (B) Frail elderly ≥ 60 in the U-PRIM or the U-PRIM+U-PROFIT group of a cluster RCT. The inclusion criteria are the same as mentioned above. | (A) Frail elderly just below 75 from the same GP practices living at home, identified by screening with U-PRIM who do not participate in U-PROFIT. The comparator group needs to meet the same frailty inclusion criteria as the intervention group.  (B) Frail elderly ≥ 60 in control group of cluster RCT not receiving U-PRIM or U-PROFIT. The comparator groups needs to meet the same inclusion criteria. |
| *Care Chain Frail Elderly (CCFE)* | Prospective quasi-experimental, PSM | Frail elderly living at home with complex care needs and loss of control, from 3 primary care groups participating in a bundled care programme for frail elderly.  Patients are identified by the GP and the nurse practitioner working in the GP practice. Quantitative inclusion criteria are not used but practices are instructed to select the frailest elderly still living at home, because the health insurer provides this bundled payment for a maximum of 1% of the GP patients. | Similar frail elderly from same region, receiving usual care from GPs of 1 the 3 primary care groups that has GPs who have not yet implemented the programme.  Patients are identified by the GP and the nurse practitioner with the help of a GP specialised in frailty (kaderarts) to ensure the inclusion of similar patients as in the intervention group. |
| *Better Together in Amsterdam North (BSiN)* | Prospective quasi-experimental, PSM | Residents of Amsterdam with limited self-sufficiency in multiple life domains referred for participation in BSiN.  Different providers from primary, secondary and social care can refer a person to be triaged. The triage is done by a team including the project leader of BSiN who is a social worker, a GP, a district nurse, and a welfare worker. Persons with a score of three or lower on at least three of the 11 life domains of the Self Sufficiency Matrix (SSM) are assigned to case management. | Residents of Amsterdam with limited self-sufficiency identified in the ‘Amsterdam Health Monitor' in 2012 and 2016.  They were identified by ‘mapping’ several questions from the AGM onto the SSM in order to decide whether persons had a decreased self-sufficiency. |
| **Norway** | | | |
| *Learning Networks* | Prospective quasi-experimental, PSM | The Norwegian association of local and regional authorities, has selected 11 municipalities of varying sizes from the Agder counties and 1 from Møre and Romsdal for the provision of the programme. These municipalities select frail elderly who are recently dismissed from hospital to care at home, from short term care at a nursing home to care at home, or live at home and have recently experienced a reduction in their health.  These municipalities have participated in 'Learning Networks’ during the period 2013-2017. | For the control group the study includes a similar group of frail elderly from municipalities who are not part of ‘Learning Networks’, i.e. who do not offer a care pathway programme. These comparator municipalities are selected from the county of Møre and Romsdal, and the city of Bergen. Patients were included based on the same inclusion criteria as were used for patients in intervention municipalities. |
| *Medically Assisted Rehabilitation (MAR) Bergen* | Prospective and retrospective quasi-experimental, PSM | People with opioid addiction participating in a programme on integrating health and social care services of specialists and the municipalities in Bergen. A criterion for participation in the treatment group was that patients were enrolled in the programme based on opioid addiction diagnosis according to ICD-10/DSM-IV, and were included in the MAR register that was established in 2017. The register provides demographic information, data on health and medical conditions of patients, their opioid use, and contacts with health care and social services. | Patients with opioid addiction from the cities of Oslo, Stavanger and Trondheim. These patients were identified in an annual national survey among MAR patients in Norway that includes data on their opioid use, demographics, life satisfaction, and contacts with the health care and legal system. They were matched to the patients in Bergen’s MAR register using demographic and medical indicators in the national survey that were similar to the indicators in the MAR register in Bergen. |
| **Spain** |  |  |  |
| *Barcelona-Esquerra (AISBE)* | (A) Retrospective quasi-experimental population-based evaluation, PSM  (B) Cross-sectional programme-component evaluation | A) Residents served by the Barcelona-Esquerra healthcare provider organizations that offer integrated care services for chronic patients across healthcare tiers in the AISBE district.  (B) Patients admitted to the hospital at home/early discharge programme offered by Hospital Clinic. Selection was done prospectively by the Integrated Care Unit team at the Emergency department, Internal Medicine and Pneumology wards (including day hospital services). Patients were included when they: i) lived in the region where the AISBE programme is offered; ii) had an informal carer during 24h per day; iii) had phone at home; and, iv) signed written acceptance to be treated at home by the Integrated Care team. Patients were excluded if they: i) lived in a nursing home; ii) had high risk of severe clinical deterioration not treatable at home, as assessed by best medical judgment; iii) were admitted in a short stay unit; iv) had severe psychiatric disorder, and, v) there was insufficient manpower of the professional team running the program for additional admissions to Home Hospitalization/Early Discharge. | (A) Residents of the entire region that are served by other provider organizations in the same region of Barcelona-Esquerra. Patient data for comparison is available through the Catalan Health Surveillance System (registry).  (B) Comparable group of patients from a comparable hospital (Hospital Sagrat Cor) that does not offer hospital at home/early discharge.  Patients were prospectively identified on an everyday basis by one of the authors (EB) after screening of the electronic medical records of the Internal Medicine and Pneumology wards. Patients were approached for informed consent if they lived in Barcelona, were admitted to the wards through the Emergency Department or day hospital service and were not previously recruited by the Integrated Care Unit team. |
| *Badalona Serveis Assistencials (BSA)* | Prospective and retrospective quasi-experimental, PSM | Individuals living in Badalona who participate in BSA’s integrated care programme for frail elderly that includes: (i) Early Discharge support; (ii) Long-term home-based support services and (iii) Residential care.  For all three groups, inclusion criteria include age > 65, 2 or more chronic diseases (COPD, congestive cardiac disease, Diabetes, hypertension, depression and anxiety disorder) and polypharmacy (≥ 4 medicines).  Specific selection criteria for group (i) being discharged form hospital after an acute disease that required continuity of care by the Home Hospitalization team). For group (ii) patients were identified as complex chronic patients or those with less than one-year life-expectancy and 3 or more emergency or hospital admissions in the last year. For group (iii) patients were recruited if they were interned in a geriatric residence served by BSA. | For each of the three intervention groups, a corresponding control group was selected among individuals living in Badalona but attended by providers or living in residencies not included in the BSA program.  Patients were selected according to the same general criteria (age, chronicity and polypharmacy) used for the three intervention groups. Specifically, group (i) included patients discharged from the Hospital Municipal de Badalona (HMB) with usual follow-up by primary care teams. Group (ii) included complex chronic patients or those with less than one-year life-expectancy and 3 or more emergency or hospital admissions in the last year who did not enter a case management programme. For group (iii) the patients were recruited from geriatric residences served by the Institut Catala de la Salut (ICS) in Badalona, Mongat and Tiana. |
| **UK** |  |  |  |
| *Salford Integrated Care Programme (SICP) / Salford Together* | (A) Retrospective quasi-experimental population-based evaluation; difference-in-differences analyses (using matching), exploiting gradual roll-out and geographical limits, and examining differential effect by multi-morbidity status.  (B) Retrospective quasi-experimental programme-component evaluation | A) Individuals 65+ with long-term conditions that are eligible for the following 3 services by 1 clinical commissioning group, i.e., case management, community groups, a centralised telephone hub to help with navigating.  On initial roll-out of the programme, the over 65 group were the primary targets. The programme is a population health management one, so targets healthy to high-risk individuals with the range of interventions.  (B) Individuals 65+ receiving case-management. The programme initially targeted high-risk (of secondary care utilisation) individuals via a risk tool. We additionally focus on a multi-morbid subgroup, those most likely to be directly treated. | A) Entire population of 65+ in England and populations of 65+ from other geographical regions (i.e., other clinical commissioning groups not offering a similar integrated care programme) and other time periods.  This data is available in large nationally representative datasets on utilisation and patient experience in England.  (B) Salford population 65+ with similar multi-morbidity not receiving case management. We exclude patients from other ‘vanguard’ programmes in England, those most likely to be receiving a similar intervention. |
| *South Somerset Symphony Programme (SSSP)* | (A) Retrospective quasi-experimental population-based evaluation; difference-in-differences analyses (using matching if necessary), exploiting gradual roll-out and geographical limits, and examining differential effect by multi-morbidity status.  B) Retrospective quasi-experimental programme-components evaluation | (A) Population of the Clinical Commissioning Group that offers the SSSP including complex care hubs of GPs in the hospital and co-location of health coaches in all GP practices.  On initial roll-out of the programme, those with three or more of a selection of chronic diseases were the primary targets. The programme is a population health management one, so targets healthy to high-risk individuals with the range of interventions.  (B) i) Individuals using the complex care hubs, i.e. the highest-risk (of secondary care utilisation) patients.  ii) Individuals in GP practices incorporating health coaches (enhanced primary care) as this was gradually rolled out in three waves. The gradual roll-out allows for a natural experiment. | (A) Entire population of England and other geographical regions and other time periods. This data is available in large nationally representative datasets on utilisation and patient experience in England.  (B) (i) Propensity matched persons within South Somerset not using the complex care hubs. There is a specific capacity of patients that can be treated by the hub, and patients at similar risk levels that are not (immediately) treated.  (ii) Practices act as controls until they roll-out the intervention, allowing us to determine short-term effects of the health coaching intervention on the practice population and specific subgroups. |
