# Supplementary material for: Strengthening the evidence-base of integrated care for people with multi-morbidity in Europe using Multi-Criteria Decision Analysis (MCDA)
Source: BMC Health Serv Res. 2018 Jul 24;18:576. doi: 10.1186/s12913-018-3367-4 (PMC6057041; doi:10.1186/s12913-018-3367-4)
Supplement: Supplementary file 3 — Table S3. Definition of outcome criteria (attributes) and levels in the DCE. (DOCX 14 kb) [file 12913_2018_3367_MOESM3_ESM.docx]

Table S3. Definition of outcome criteria (attributes) and levels in the DCE.

| **Outcome criteria** | **Definition** | **Attribute-levels** |
| --- | --- | --- |
| **Physical functioning** | Acceptable physical functioning and being able to do daily activities without needing assistance (e.g., getting dressed, sitting down and getting up from a chair, taking your medications) | 1. Severely limited in physical functioning and activities of daily living  2. Moderately limited in physical functioning and activities of daily living  3. Hardly or not at all limited in physical functioning and activities of daily living |
| **Psychological well-being** | The absence of stress, worrying, listlessness, anxiety, and feeling down | 1. Always or mostly stressed, worried, listless, anxious, and down  2. Regularly stressed, worried, listless, anxious, and down  3. Seldom or never stressed, worried, listless, anxious, and down |
| **Social relationships and participation** | Having meaningful connections with others as desired | 1. No or barely any meaningful connections with others  2. Some meaningful connections with others  3. A lot of meaningful connections with others |
| **Enjoyment of life** | Having pleasure and happiness in life | 1. No or barely any pleasure and happiness in life  2. Some pleasure and happiness in life  3. A lot of pleasure and happiness in life |
| **Resilience** | The ability to recover from or adjust to difficulties and to restore one’s balance | 1. Poor ability to recover, adjust, and restore balance  2. Fair ability to recover, adjust, and restore balance  3. Good ability to recover, adjust, and restore balance |
| **Person-centeredness** | Care that matches persons’ needs, capabilities, and preferences and where decisions are made jointly based on good information | 1. Not or barely person-centred  2. Somewhat person-centred  3. Highly person-centred |
| **Continuity of care** | Good collaboration, smooth transitions between caregivers, and no waste of time | 1. Poor collaboration, transitions, and timeliness  2. Fair collaboration, transitions, and timeliness  3. Good collaboration, transitions, and timeliness |
| **Total health- and social care costs** | The total health- and social care costs per participant in the programme, per year. Note: These are costs paid for by the health insurer / government | 1. 8500 euros per participant per year*  2. 7000 euros per participant per year*  3. 5500 euros per participant per year* |

*These are figures for the Netherlands; they differ between countries, but in all countries the highest and lowest level were defined as + or – 20% of the middle level
