# Supplementary material for: Strengthening the evidence-base of integrated care for people with multi-morbidity in Europe using Multi-Criteria Decision Analysis (MCDA)
Source: BMC Health Serv Res. 2018 Jul 24;18:576. doi: 10.1186/s12913-018-3367-4 (PMC6057041; doi:10.1186/s12913-018-3367-4)
Supplement: Supplementary file 4 — Table S4-S7. Supplementary outcome criteria and their worst and best levels in Swing Weighting. (DOCX 19 kb) [file 12913_2018_3367_MOESM4_ESM.docx]

**Supplementary outcome criteria used in Swing Weighting**

**Table S4. Supplementary set of outcome criteria for Population Health Management programmes and their worst (red) and best (green) level**

| **Activation & engagement**   - Defined as taking on the role of managing one’s own health and care   **Unsuccessful in** managing own health and care  **Actively** managing own health and care |
| --- |
| **Avoidable hospital admissions**   - Defined as the number of hospital admissions that could have been avoided with better care   **15 out of 100** hospital admissions could have been avoided with better care  **5 out of 100** hospital admissions could have been avoided with better care |
| **Hospital re-admissions**   - Defined as the number of persons who are re-admitted to a hospital within 30 days of their prior hospital discharge.   **10 out of 100** participants are re-admitted to hospital within 30 days of hospital discharge.  **5 out of 100** participants are re-admitted to hospital within 30 days of hospital discharge. |

**Table S5. Supplementary set of outcome criteria for Frail Elderly Programmes and their worst (red) and best (green) level**

| **Autonomy**   - Defined as remaining in charge and making own decisions on how one lives his/her own life   **Not, or barely**, in charge of one’s own life nor making own decisions  **Fully** in charge of one’s own life and making own decisions |
| --- |
| **Burden of medication**   - Defined as the amount of burden medicines are causing, considering, for example, administering/taking the medicines, side effects, understanding their purpose and why they’re being taken, worries about interaction between medicines, and expenses   **High** burden of medication  **No or low** burden of medication |
| **Burden of informal caregiving**   - Defined as the stress of informal caregiving due to the energy it costs, the little time it leaves for own interests and recovery and the sadness over the fate of the supported person   **High** burden of informal caregiving  **No or low** burden of informal caregiving |
| **Long-term institution admissions**   - Defined as the number of participants admitted to long-term institutional care (for example a nursing home), during the programme   **20 out of 100** participants are admitted to a long-term institution  **10 out of 100** participants are admitted to a long-term institution |
| **Falls leading to hospital admissions**   - Defined as the number of participants admitted to an emergency room or hospital because of a fall   **10 out of 100** participants have a fall that results in a hospital admission  **5 out of 100** participants have a fall that results in a hospital admission |

**Table S6.** **Supplementary set of outcome criteria for Oncological and Palliative Care programmes and their worst (red) and best (green) level**

| **Life expectancy**   - Defined as the change in 3-month life expectancy.   **No improvement** in life expectancy  **Improvement** in life expectancy |
| --- |
| **Pain and other symptoms**   - Defined as physical symptoms like pain, fatigue, nausea/vomiting, short of breath, appetite loss, constipation/diarrhoea, insomnia   **A lot of** pain and symptoms  **Little, or no,** pain and symptoms |
| **Burden of informal caregiving**   - Defined as the stress of informal caregiving due to the energy it costs, the little time it leaves for own interests and recovery and the sadness over the fate of the supported person   **High** burden of informal caregiving  **No, or low,** burden of informal caregiving |
| **Compassionate care**   - Defined as care that is provided in a warm, sensitive and dignified way with sympathy and respect   **Not, or barely,** compassionate  **Very** compassionate |
| **Timely access to care**   - Defined as the time between referral and start of treatment or care of interest   **A long** time between referral and start of treatment/care  **A short** time between referral and start of treatment/care |
| **Preferred place of death**   - Defined as the number of persons that pass away in the location of their preference (e.g., at home).   **50 out of 100** participants pass away in their preferred location  **75 out of 100** participants pass away in their preferred location |

**Table S7. Supplementary set of outcome criteria for programmes that target persons with problems in multiple life domains and their worst (red) and best (green) level**

| **Financial independence**   - Defined as being financially in control to meet basic needs with little or no debts.   Financially **not in control** with growing debts  Financially **in control** and finances well-managed |
| --- |
| **Contact with justice system**   - Defined as contact with justice system, such as with criminal justice services, nights in police cell, and court attendance   **Regular** contact with justice system  **No or rare** contact with justice system |
